# Impact of BNT162b2 primary vaccination and homologous booster on anti-SARS-CoV-2 IgA antibodies in baseline seronegative healthcare workers

**DOI:** 10.1515/almed-2022-0033

**Published:** 2022-05-25

**Authors:** Gian Luca Salvagno, Brandon M. Henry, Laura Pighi, Simone De Nitto, Giuseppe Lippi

**Affiliations:** Section of Clinical Biochemistry, University of Verona, Verona, Italy; Service of Laboratory Medicine, Pederzoli Hospital, Peschiera del Garda, Italy; Clinical Laboratory, Division of Nephrology and Hypertension, Cincinnati Children’s Hospital Medical Center, Cincinnati, OH, USA

**Keywords:** antibodies, COVID-19, IgA, SARS-CoV-2, vaccination

## Abstract

**Objectives:**

We investigated here the response of anti-SARS-CoV-2 IgA antibodies to BNT162b2 primary vaccination followed by administration of a homologous booster dose in baseline seronegative healthcare workers.

**Methods:**

The study population included 69 healthy recipients of primary BNT162b2 vaccination (two doses) followed by administration of a single homologous booster after 8 months. Blood samples were collected throughout the study, starting before the first vaccine dose, up to 1 month after the booster. The serum levels of anti-SARS-CoV-2 IgA were assayed with Euroimmun Anti-SARS-CoV-2 spike S1 ELISA IgA.

**Results:**

A first peak of serum anti-SARS-CoV-2 IgA was seen 1 month after the second BNT162b2 dose, after which values gradually declined, with stabilization after 6 months. The BNT162b2 booster (third dose) elicited a second peak, comparable to that observed 1 month after the second dose (p=0.100). Highly significant correlation was found between pre- and post-booster anti-SARS-CoV-2 IgA serum values (r=0.41; p<0.001), whilst no significant correlation was observed with age (r=0.10; p=0.416) or sex (r=0.04; p=0.729). The rate of SARS-CoV-2 IgA seropositive recipients increased from 0% before vaccination to 80 and 97% after the first and second vaccine dose, but then declined becoming 74% at 3 months and 54% at 6 months, respectively, after which stabilization was reached. The BNT162b2 booster dose restored the seropositivity rate to 99%.

**Conclusions:**

These results support the suggestion that vaccine boosters may be advisable after 3 months from primary vaccination to restore IgA to protective levels, especially in those at higher risk of SARS-CoV-2 infection and complications.

## Introduction

Although some physical measures may be effective to limit the worldwide burden of coronavirus disease 2019 (COVID-19) [1], unrelieved procrastination of such measures is almost unfeasible for many social, economic and even psychological reasons [2]. Widespread vaccination against severe acute respiratory syndrome coronavirus disease 2 (SARS-CoV-2) must hence be considered the most efficient strategy for preventing the risk of developing severe COVID-19 illness (requiring hospitalization, mechanical ventilation, intensive care and so forth), as well as for efficiently limiting viral circulation [3]. Nonetheless, several lines of evidence now attest that the individual response to the different formulations of COVID-19 vaccines varies broadly [4], and this is mostly reflected by heterogeneous generation of anti-SARS-CoV-2 neutralizing antibodies depending on several individual aspects such as age, sex, concomitant therapies and co-morbidities [5]. Strict longitudinal monitoring of humoral immunity and identification of significant predictors of vaccine response are hence now universally considered as a pivotal aspect for optimizing vaccine administration [6]. Among the various classes of antibodies that are elicited by vaccination, immunoglobulins A (IgA) play a crucial role, since their serum values closely reflect the development of mucosal humoral immunity, which is effective to substantially limit the risk of contagion [7].

In 2021, we started a longitudinal serosurveillance study aimed at monitoring and interpreting the kinetics of serum anti-SARS-CoV-2 antibodies developed in a cohort of healthy recipients of BNT162b2 vaccination [8]. We provide here an updated analysis of this ongoing trial, by analyzing the variation of anti-SARS-CoV-2 IgA antibodies after primary vaccination and after receiving a homologous vaccine booster.

## Materials and methods

The original study population consisted of 100 in baseline seronegative healthcare workers of the Pederzoli Hospital (Peschiera del Garda, Italy), who were baseline SARS-CoV- seronegative and underwent primary vaccination with the COVID-19 vaccine BNT162b2 (Pfizer Inc., New York, NY; two doses of 30 µg, separated by 3 weeks), followed by administration of a single homologous booster dose (30 µg) after 8 months from completing the primary vaccination cycle. Molecular assays for diagnosing incident SARS-CoV-2 infection throughout the study period were conducted at 2–4 weeks intervals with either Altona Diagnostics RealStar SARS-CoV-2 RT-PCR Kit (Altona Diagnostics GmbH, Hamburg, Germany) or Seegene Allplex SARS-CoV-2 Assay (Seegene Inc., South Korea). Venous blood sample were drawn from each subject before receiving the first and second vaccine doses of the primary vaccination cycle, whilst additional blood collections were then performed after 1, 3 and 6 months, as well as immediately before receiving the homologous BNT162b2 vaccine booster and 1 afterwards.

The serum levels of anti-SARS-CoV-2 spike S1 IgA were assayed using Anti-SARS-CoV-2 ELISA IgA (Euroimmun, Lübeck, Germany). This test is a manual enzyme linked immunosorbent assay (ELISA), whose technical and diagnostic characteristics have been thoughtfully described elsewhere [9, 10]. Briefly, the total imprecision of the method is <2%, the negative and positive predictive values are 99% and 66%, reaching 100% diagnostic sensitivity compared with a cell culture-based microneutralization test. Test results are considered positive when the value of serum IgA (expressed as ratio to the cut-off) is ≥1.1. Results of measurements were expressed as median and interquartile range (IQR), and analyzed with Mann–Whitney U and Chi-square (with Yates’ correction) tests, and with Spearman’s correlation, using Analyse-it (Analyse-it Software Ltd, Leeds, UK). All study subjects provided written informed consent for being vaccinated and for undergoing anti-SARS-CoV-2 antibodies monitoring. This observational study was reviewed and cleared by the Ethics Committee of Verona and Rovigo provinces (59COVIDCESC; November 3, 2021), and was conducted in accordance with the Declaration of Helsinki, under the terms of relevant local legislation.

## Results

**Figure 1:**
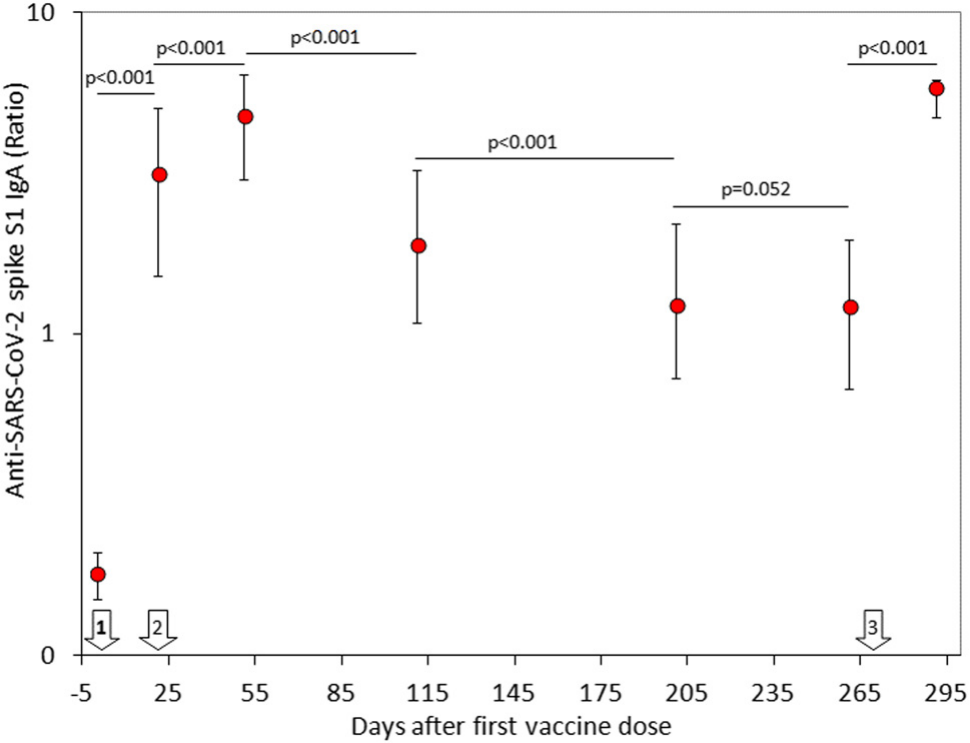
Serum concentration (median and interquartile range) of anti-SARS-CoV-2 spike S1 IgA antibodies in a cohort of healthcare workers receiving primary BNT162b2 vaccination and homologous booster. The white arrows indicate the timing of BNT162b2 vaccine doses.

The final study population consisted of 69 in baseline seronegative healthcare workers (median age, 44 years; IQR, 32–52 years; 38 females), since 31 subjects were lost during the follow-up for missing one or more vaccine doses, failing to have their blood collected at one or more time points, or testing positive for SARS-CoV-2 RNA during the study. The variation of anti-SARS-CoV-2 spike S1 IgA throughout the study period is shown in Figure 1. A first peak was clearly appreciable 1 month after the second BNT162b2 dose, whilst the serum values of the antibodies displayed a gradual decline over time. Notably, a relative stabilization at low levels was achieved over 8 months after the second vaccine dose, as anti-SARS-CoV-2 spike S1 IgA values were no longer significantly different from those measured after the second vaccine dose (p=0.100). The administration of the BNT162b2 booster (third dose) generated a second peak of serum anti-SARS-CoV-2 spike S1 IgA antibodies, which was not significantly different from the values observed 1 month after the second vaccine dose (Figure 1). A highly significant correlation was found between pre- and post-booster anti-SARS-CoV-2 spike S1 IgA serum values (r=0.41; 95% CI, 0.19–0.59; p<0.001) (Figure 3), whilst no correlation was observed between post-booster anti-SARS-CoV-2 spike S1 IgA serum values and age (r=0.10; 95% CI, −0.14 to 0.33; p=0.416) or sex (r=0.04; 95% CI, −0.20 to 0.28; p=0.729).

**Figure 2:**
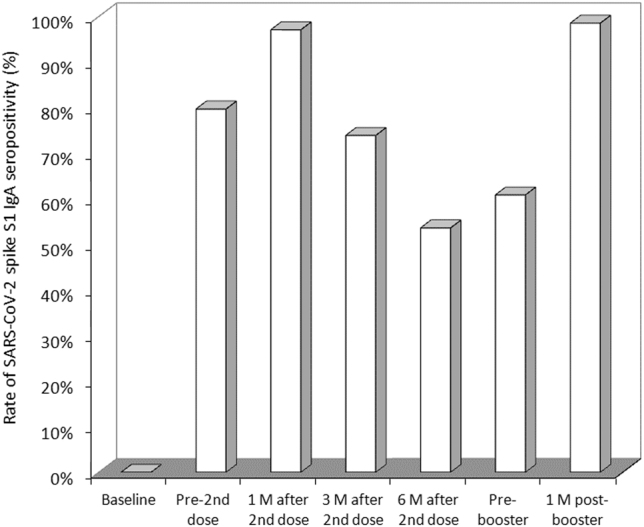
Rate of SARS-CoV-2 spike S1 IgA seropositivity in healthcare workers receiving primary BNT162b2 vaccination and homologous booster. M, months.

The rate of vaccine recipients with anti-SARS-CoV-2 spike S1 IgA values above the method-dependent cut-off (i.e., ≥1.1) is shown in Figure 2. As predictable, the rate increased from 0% before vaccination to 80 and 97% after receiving the first and second vaccine dose, respectively. In keeping with the serum levels, the rate of seropositive subjects also declined over time, becoming 74% at 3 months and 54% at 6 months, after which it stabilized at around 60%. The administration of the BNT162b2 booster (third dose) was effective to restore the SARS-CoV-2 IgA seropositivity rate up to 99%.

**Figure 3:**
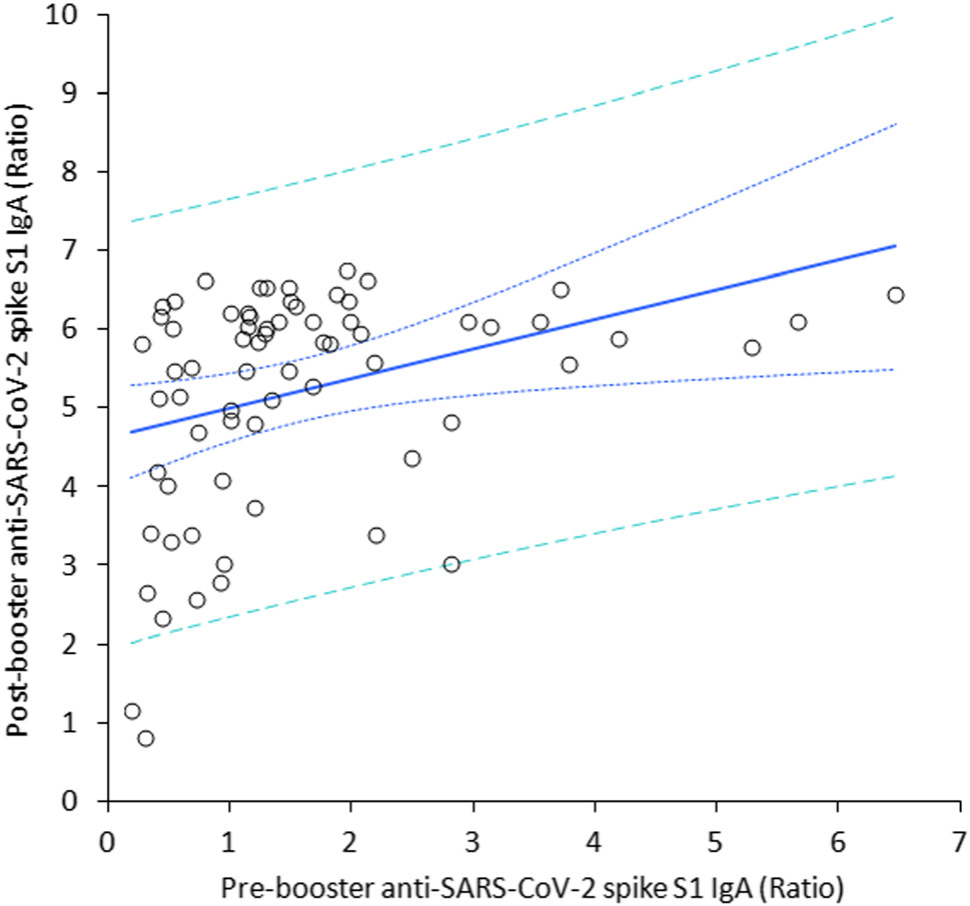
Spearman’s correlation between the serum concentration of anti-SARS-CoV-2 spike S1 IgA antibodies before and after receiving a BNT162b2 vaccine booster.

## Discussion

In this study we have aimed to provide a reliable picture of anti-SARS-CoV-2 IgA kinetics after BNT162b2 primary vaccination and administration of a homologous booster dose in ostensibly healthy healthcare workers. Several important aspects have emerged.

We have first demonstrated that the variation of serum anti-SARS-CoV-2 IgA partially mirrors that of serum anti-SARS-CoV-2 spike trimeric IgG antibodies [11]. Nonetheless, unlike IgG, we found that the serum levels of IgA seemingly display a milder decline over time, reaching stabilization at low levels between 6 and 8 months after completing the primary vaccination cycle. Similarly, the rate of seropositivity also declined over time, but remained relatively stable (at around 60%) up to 8 months after primary vaccination. This seems at odds with data obtained measuring anti-SARS-CoV-2 spike trimeric IgG antibodies, whose rate of protective levels continuously declines over time. Nonetheless, the relatively high number of SARS-CoV-2 IgA seronegative subjects already identified 6 months after primary vaccination supports the opportunity of vaccine booster administration, especially in the more fragile parts of the population, which may be more vulnerable to unfavorable disease progression, as well as in those who had become seronegative over time.

We also found that the pre-booster serum levels of anti-SARS-CoV-2 spike S1 IgA significantly predicted the post-booster values, whilst no significant association was found with age and sex. This suggests that subjects with milder decline of anti-SARS-CoV-2 IgA may be those who tend to show better response to the homologous vaccine booster. This is important for optimizing COVID-19 vaccines administration. Due to the current challenges in ensuring universal COVID-19 vaccination coverage [12], especially in underdeveloped countries in which vaccine availability is dramatically limited, personalizing vaccination (in terms of both dosage and timing) would allow to save precious economic and human resources. A difference was also observed with the trend of anti-SARS-CoV-2 spike trimeric IgG antibodies, whose second peak at 1 month after the vaccine booster was found to be nearly threefold higher compared to values seen after the first peak [11], whilst the two peaks reached by anti-SARS-CoV-2 IgA after the second and the third vaccine dose were globally comparable. Importantly, considering the important function played by serum IgA antibodies in protecting the organism against SARS-CoV-2, in that they reliably mirror the development of mucosal humoral immunity [7], we believe that their measurement shall complement serosurvey studies aimed at monitoring humoral response after administering COVID-19 vaccines and boosters.

In conclusion, considering the high correlation that exists between serum and secretory anti-SARS-CoV-2 neutralizing IgA [13], the results of this updated analysis of our serosurveillance trial support further the suggestion that a vaccine booster may be advisable after 3 months from primary vaccination to restore IgA to protective levels, especially in those at higher risk of SARS-CoV-2 infection and complications [14].
